# Editorial

**DOI:** 10.1155/EDR.2000.i

**Published:** 2000

**Authors:** Anders A. F. Sima, Eleazar Shafrir


*Int. Jnl. Experimental Diab. Res.*, Vol. 1, p. i
Reprints available directly from the publisher
Photocopying permitted by license only

© 2000 OPA (Overseas Publishers Association) N.V.
Published by license under
the Harwood Academic Publishers imprint,
part of The Gordon and Breach Publishing Group.
Printed in Malaysia.

### Editorial

The incidence of diabetes has been increasing alarmingly during recent decades. This is true both for Type I diabetes, which is estimated to increase world-wide by 3% per annum, and for Type II diabetes, which has become a global epidemic. The latter is a lifestyle disorder related to increasing affluence in nutrition in newly industrialized countries. These circumstances mandate extensive research into the pathogenesis of diabetes and its complications as well as their prevention. There is indeed an increase in investigative effort on topics related to diabetes. However, with limitations of space available for publication, even excellent papers are not being accepted for publication and worthwhile communications can take too long before they are published. Also, strong competition between clinical and basic science oriented papers is a delaying factor. These considerations led us to start the *International Journal of Experimental Diabetes Research*. We intend to publish results of original research on physiology, pathobiology and immunology of diabetes and related relevant topics both in experimental animal models and *in vitro* systems. We also welcome research into prevention and therapy of diabetes and its complications. It goes without saying that animals of various types and particular aspects of diabetes have contributed enormously to the understanding of the disease and its treatment. The numerous available models demonstrate ongoing pathological processes, immune derangements, insulin signaling abnormalities, nutritional and environmental influences. Reports on diabetes complications in various tissues, derangements of pancreatic islet function and intermediary metabolism are welcome. Papers on the mechanism of action of hormones, and pharmacological modalities targeted for human use as well as communications on new models of diabetes and investigations of their particular aberrations are also welcome. *The International Journal of Experimental Diabetes Research* will publish both regular papers, review articles and rapid communications which, if deemed important, will receive a rapid turn-around. We therefore invite all members of the multidisciplinary diabetes research community to submit their research findings to this Journal and become part of the team coping with the field of diabetes in the new century.

*Anders A. F. Sima*
*Eleazar Shafrir*

